# Pan-cancer landscape of UBD/FAT10 and experimental validation in esophageal carcinoma

**DOI:** 10.3389/fonc.2025.1615898

**Published:** 2025-11-19

**Authors:** Xu Zhang, Hongnian Pan, Xiuzhong Wang

**Affiliations:** 1 Department of Gastroenterology, Lu’an Hospital of Anhui Medical University, Lu’an, China; 2 Department of Gastroenterology, Lu’an People’s Hospital of Anhui Province, Lu’an, China

**Keywords:** ubiquitin D, cancer, TCGA, immune, pan cancer

## Abstract

**Objective:**

To comprehensively characterize the pan-cancer roles of Ubiquitin D (UBD/FAT10) in tumorigenesis, immune regulation, and therapeutic response through integrative multi-omics and expe+rimental analyses.

**Methods:**

Utilizing bulk RNA-seq (TCGA/GTEx/CPTAC), immune deconvolution, proteomics, and functional enrichment, we analyzed UBD expression, survival prognosis, immune infiltration, and molecular pathways across 33 cancers. Molecular docking and MD simulations were performed to assess UBD-protein interactions. Through lentivirus-mediated overexpression, functional assays (CCK-8, colony formation, wound healing, and Transwell), transcriptome sequencing, and biochemical validation, we demonstrated that UBD promotes malignant phenotypes in esophageal cancer via the TP53 signaling pathway.

**Results:**

UBD was upregulated in 14 cancers but downregulated in thyroid carcinoma (THCA) and kidney chromophobe (KICH). ROC analysis highlighted UBD’s diagnostic potential (AUC >0.8 in gastrointestinal tumors). High UBD conferred protection in melanoma (SKCM, HR = 0.891) and sarcoma (SARC, HR = 0.899) but predicted poor outcomes in uveal melanoma (UVM, HR = 1.298) and pancreatic adenocarcinoma (PAAD, HR = 1.143).UBD positively correlated with the IFN-γ-dominant immune subtype (C2), characterized by CD8+ T cells/M1 macrophages. Drug sensitivity profiling nominated imatinib (Vina score: -8.9 kcal/mol) and TTNPB as potential therapies for UBD-high tumors, validated by stable MD simulations. In esophageal carcinoma (ESCA), UBD expression escalated with tumor stage and predicted poor survival (p<0.05).UBD enhances the proliferation and migration of esophageal cancer cells by modulating the TP53 signaling pathway, as validated through transcriptomic analysis and functional assays.

**Conclusions:**

This study advances UBD as a prognostic indicator and therapeutic target, bridging molecular insights with clinical translation in precision oncology.

## Introduction

The ubiquitin-proteasome system (UPS) is a cornerstone of cellular protein homeostasis, orchestrating the degradation of damaged or regulatory proteins to maintain genomic stability, cell cycle progression, and immune surveillance (1). Among UPS components, ubiquitin-like modifiers (UBLs) have emerged as critical regulators of substrate specificity, with Ubiquitin D (UBD/FAT10) representing a unique cytokine-inducible UBL that bridges inflammation, immunity, and cancer (2, 3). Unlike canonical ubiquitin, UBD directly tags substrates for proteasomal degradation without forming polyubiquitin chains, a feature that underscores its distinct role in both physiological and pathological contexts (4).

UBD is encoded within the major histocompatibility complex (MHC) locus on chromosome 6p21.3, a genomic region densely populated with immune-related genes (5). Its expression is tightly regulated by pro-inflammatory cytokines such as interferon-γ (IFN-γ) and tumor necrosis factor-α (TNF-α), positioning UBD as a molecular nexus linking chronic inflammation to oncogenesis (6). Mechanistically, UBD drives genomic instability by destabilizing tumor suppressors (e.g., p53) and promotes immune evasion through modulation of MHC-I antigen presentation (7). Paradoxically, UBD overexpression also induces apoptosis in specific cellular contexts, suggesting a dual role contingent on tumor microenvironment (TME) dynamics (8). Despite these advances, existing studies remain fragmented, focusing predominantly on isolated cancer types (e.g., hepatocellular carcinoma, colorectal cancer), while a systematic pan-cancer analysis of UBD’s clinical relevance, immune interactions, and molecular mechanisms remains elusive (9).

The advent of multi-omics bioinformatics platforms offers unprecedented opportunities to dissect UBD’s roles across malignancies. Public repositories such as The Cancer Genome Atlas (TCGA), Genotype-Tissue Expression (GTEx), and Clinical Proteomic Tumor Analysis Consortium (CPTAC) provide comprehensive datasets spanning genomic, transcriptomic, proteomic, and clinical dimensions (10). Leveraging these resources, we aim to elucidate the pan-cancer landscape of UBD dysregulation and its prognostic significance, the interplay between UBD expression, immune cell infiltration, or immunotherapy response, as well as the potential biological pathways involving UBD.

This study represents the first integrative pan-cancer analysis of UBD, combining bulk and RNA sequencing, immune deconvolution algorithms, and functional enrichment analyses. We further validate key findings using *in vitro* models to elucidate UBD’s role in cancer cell. Our results not only delineate UBD as a potential biomarker for tumors with IFN-γ Dominant(C2)immune subtype but also highlight its therapeutic potential in cancers. By bridging molecular insights with clinical applicability, this work advances our understanding of UBD’s multifaceted contributions to oncogenesis and paves the way for targeted therapeutic strategies.

## Materials and methods

### Expression of UBD in pan-cancer

We obtained a uniformly standardized pan-cancer dataset (TCGA, GTEx) and retrieved UBD gene expression data across tumor and normal tissues from the UCSC Xena Browser (http://xenabrowser.net/) (11). Mutational profiles, copy number alterations (CNAs), and gene fusion events were analyzed using the cBioPortal platform (https://www.cbioportal.org/) based on the TCGA Pan-Cancer dataset (12). Additionally, gene-level Copy Number Variation (CNV) data and Level 4 gene expression profiles for all TCGA samples, processed via GISTIC software (DOI: 10.1186/gb-2011-12-4-r41), were downloaded and integrated from the GDC portal (https://portal.gdc.cancer.gov/) (13, 14). Differential UBD expression between tumor and normal tissues was assessed using R software. Raw expression matrices underwent log2(x + 0.001) transformation to stabilize variance, followed by batch effect correction using the ComBat-seq algorithm. Statistical significance was defined as P < 0.05.

### Pan - cancer survival analysis of UBD expression

The Kaplan–Meier (KM) survival analysis technique is a commonly - used statistical method for comparing survival differences among diverse cohorts. In the present study, we utilized the survival package within R to carry out KM survival analysis on patient groups with high and low UBD expression across 33 cancer types, which covered overall survival (OS), disease - specific survival (DSS), and progression - free interval (PFI) (31). The Cox regression model was applied to calculate p - values and assess the statistical significance of survival differences. Through the use of the Survminer and ggplot2 packages, we computed hazard ratios (HRs), 95% confidence intervals (CIs), and p - values, and presented these results visually. The KM survival analysis utilized the “surv_cutpoint” function from the R package survminer to determine the optimal cutpoint for continuous variables. This function calculates maximally selected rank statistics based on the maxstat package, and identifies the optimal cutoff value by maximizing this statistic to define the most discriminative grouping.

### Association analysis of UBD with tumor immune cell infiltration

We systematically evaluated the influence of UBD expression levels on the extent of immune cell infiltration across a diverse array of cancer types within the TCGA database using seven methods, namely XCELL, CiberSort_ABS, CiberSort, EPIC, QUANTISEQ, MCPCOUNTER, and TIMER. The R packages utilized for quantifying immune infiltration included CIBERSORT, xCell, IOBR, MCPcounter, and the quanTIseq package (15).

The study “The Immune Landscape of Cancer” conducted a large-scale immunogenomic analysis of over 10,000 tumor samples from 33 different cancer types available in the TCGA database (16). In this pan-cancer analysis, researchers identified six distinct immune subtypes based on the following criteria: macrophage or lymphocyte markers, the ratio of Th1 to Th2 cells, the range of tumor intergenetic heterogeneity, aneuploidy, neoantigen burden, the overall cell atlas, the expression of immune regulatory genes, and prognosis. The six subtypes are described as follows:

C1 (Wound Healing): Elevated expression of angiogenesis-related genes, high proliferative fraction, and Th2-skewed adaptive immune infiltration. C2 (IFN-γ Dominant): Highest M1/M2 macrophage polarization, strong CD8+ T cell signaling, and similar to C6, the highest T cell receptor (TCR) diversity. C3 (Inflammatory): Elevated Th17 and Th1 gene expression, inability to effectively restrain tumor cell proliferation, and like C5, fewer aneuploidies and overall copy number alterations compared to other subtypes. C5 (Immune Silent): The least lymphocytic infiltration, highest macrophage response, and M2 macrophage predominance. C6 (TGF-β Dominant): A smaller group with the highest TGF-β signature and high lymphocytic infiltration. It has an equal distribution of type I and type II T cells.

### Alterations in somatic genomic copy number and mutations of UBD

Data on somatic variants and DNA copy number alterations (CNA) for a pan - cancer analysis were obtained from the cBioPortal website (17). The Spearman correlation between UBD expression levels and DNA copy number alterations was calculated to evaluate the association between somatic copy number alterations (SCNA) and UBD expression. The results were presented in the form of a heatmap.

### Investigating the possible biological roles of UBD in Pan - Cancer

Patients from the TCGA dataset were grouped into high and low UBD expression groups according to their UBD expression levels. Gene Set Enrichment Analysis (GSEA) was used to assess the modulation of Hallmark gene sets and KEGG pathway gene sets across different expression levels in various tumors (18). Additionally, the relationship between UBD mRNA levels and protein expression measured by Reverse Phase Protein Array (RPPA) in the TCPA database was evaluated using Rank - based association analysis. The results across all tumors were visualized by heatmaps (19).

### The z - score evaluation of the biological process

The z - score algorithm proposed by Lee et al. was used to reflect the activity of specific pathways by integrating the expression of feature genes (20). Gene sets containing genes related to Angiogenesis, Epithelial - to - Mesenchymal Transition (EMT), Cell Cycle, Apoptosis, Hypoxia, Inflammation, Invasion, Metastasis, Proliferation, Quiescence, Stemness, Differentiation, DNA Damage, and DNA Repair were subjected to the z - score algorithm implemented in the GSVA R package. These gene sets related to the aforementioned tumor pathways were derived from the Cancer Single - cell Atlas (CancerSEA) database (21).

### Real-time quantitative polymerase chain reaction, Western blot

Total RNA was extracted using TRIzol reagent (Ambion, USA) following standard protocols. cDNA synthesis was performed using PrimeScript™ RT Master Mix (Takara, Japan) according to the manufacturer’s instructions. Gene expression analysis was conducted via real-time PCR with ChamQ SYBR qPCR Master Mix (Vazyme, China), with the 2^-ΔΔCT method applied for relative quantification, using GAPDH as the endogenous control.

Specific primer sequences were designed as follows:

UBD: Forward 5′-CCGTTCCGAGGAATGGGATTT-3′, Reverse 5′-GCCATAAGATGAGAGGCTTCTCC-3′. GAPDH: Forward 5′-AACAGCCTCAAGATCATCAGC-3′, Reverse 5′-GGATGATGTTCTGGAGAGCC-3′.

Three independent experimental replicates were performed to ensure data reliability, with cycle threshold (Ct) values averaged across technical triplicates. Quantitative measurements of target gene expression were normalized against the housekeeping gene GAPDH to account for potential variations in RNA input.

Protein extraction was performed using RIPA lysis buffer. Protein concentration determination was conducted with a BCA protein assay kit (Beyotime Biotechnology) following standard protocols. Electrophoretic separation was carried out on 10% SDS-PAGE with 50 μg protein samples loaded per well. After transferring the resolved proteins onto PVDF membranes, blocking was achieved with 5% non-fat milk in TBS-T to minimize background signals.

Primary antibodies included:

Rabbit anti-UBD polyclonal antibody (Thermofisher, USA;1:2000).
Rabbit anti-GAPDH polyclonal antibody (Abcam, USA;1:5000).
Rabbit anti-p53 polyclonal antibody (Proteintech, China; 1:800).
Rabbit anti-p21 polyclonal antibody (Proteintech, China; 1:1500).
Rabbit anti-cyclinB1 polyclonal antibody (Abcam, USA; 1:1000).
Rabbit anti-CDK1 polyclonal antibody (Proteintech, China; 1:1200).
Rabbit anti-CDK4 polyclonal antibody (Proteintech, China; 1:2000).
Rabbit anti-c-myc polyclonal antibody (Proteintech, China; 1:1500).

Membranes were incubated with HRP-conjugated goat anti-rabbit IgG secondary antibody (Abcam;1:5000) at 4°C for 60 minutes after thorough TBS-T washing. Protein band visualization was achieved using an enhanced chemiluminescence detection system, which optimizes the sensitivity of chemiluminescent reactions through substrate optimization.

### Colony formation assay, cell counting kit-8 assay, transwell migration assay and scratch assay

The esophageal cancer cell line TE-11 was obtained from Shanghai Fuheng Biological Technology Co., Ltd. The TE-11 cell line was maintained in Dulbecco’s Modified Eagle’s Medium (Gibco, Grand Island, USA) containing 10% fetal bovine serum (Cyagen, Suzhou, China).

For the assessment of cell proliferation and colony-forming potential, a Colony Formation Assay was conducted. Logarithmically growing cells stably expressing the gene of interest were seeded at 500 cells/mL in 6-well plates, with 1 mL of cell suspension per well, and cultured under 37°C, 5% CO2. The medium was refreshed every two days based on color change to maintain nutrient supply. After 7 days, colonies were fixed with 4% paraformaldehyde, stained with 0.1% crystal violet, and quantified using ImageJ for colony number and area analysis.

Cell proliferation and viability were assessed via Cell Counting Kit-8 (CCK-8) assay. Cells were seeded at 1*10^3 cells/well in 96-well plates and incubated at 37°C, 5% CO2. Over five days, CCK-8 solution was added daily, followed by a 1-hour incubation, and absorbance measured at 450 nm using a microplate reader.

Transwell migration assays were performed to evaluate cell migration capabilities. Cells were suspended at 2x10^5/mL in 1% FBS medium, and 100 μL of this suspension was added to Transwell chambers prehydrated with serum-free medium. Chambers were placed in 24-well plates containing 600 μL of 20% FBS medium. After 24 hours, cells were fixed with methanol, stained with 0.1% crystal violet, and non-migrated cells were gently removed. Images were captured under a microscope.

Scratch assays were utilized to investigate cell migration dynamics. Cells were plated at 6x10^5 cells/well in 6-well plates and allowed to grow until >90% confluence. Post serum-starvation for 4 hours, scratches were made with a 200 μL pipette tip, washed with PBS, and maintained in serum-free medium for 24 hours. Wound closure was monitored and analyzed using ImageJ.

### Immunohistochemistry (IHC)

This retrospective cohort study was approved by the Institutional Ethics Committee of Lu’an People’s Hospital (Ethics Approval No. 2023LLKS012). Formalin-fixed paraffin-embedded (FFPE) tissue blocks were systematically collected from patients with ESCA who underwent curative resection between January 2019 and August 2025. The inclusion criteria were as follows: histologically confirmed primary esophageal carcinoma, tumor cellularity of at least 30% in representative sections, and complete clinicopathological records. Exclusion criteria included receipt of neoadjuvant therapy and insufficient tissue for comprehensive analysis. Detailed patient characteristics are provided in **Supplementary Table 1**. Immunohistochemical analysis of UBD expression was performed using Rabbit anti-UBD polyclonal antibody (Thermofisher, #PA5-102790;1:2000). Automated optical density (AOD) values were calculated as the ratio of positive staining area to total tissue area using ImageJ software (National Institutes of Health, v1.53). Five randomly selected 400× fields per specimen. Patients were stratified into three distinct prognostic groups based on AOD tertile distribution.

### Connectivity map analysis

The LIMMA differential analysis identified the top 500 most up- or down-regulated genes between UBD-high and UBD-low groups across different cancer types, which were used as a UBD-associated gene signature. An RData file containing 1,288 compound-related signatures was downloaded from the database website (https://www.pmgenomics.ca/bhklab/sites/default/files/downloads) for matching score calculation. The analytical procedure followed methods outlined in previous publications (1, 22, 23). The results across 31 cancer types were summarized and visualized using the pheatmap package in R.

### Molecular docking and molecular dynamics simulations

The CB-Dock2 web server (https://cadd.labshare.cn/cb-dock2), which employs AutoDock Vina’s algorithm, was utilized for molecular docking studies. Default parameters were applied throughout the simulations. Drug molecular structures were obtained from PubChem (https://pubchem.ncbi.nlm.nih.gov/), and the protein target was downloaded from RCSB PDB (https://www.rcsb.org/).

MD simulations were performed using GROMACS 2022. Force field parameters were obtained using the pdb2gmx tool in GROMACS and the AutoFF web server. The CHARMM36 force field was applied to the receptor protein, while the CGenFF force field was used for the ligand molecules. The system was solvated with a cubic TIP3P water box with a margin of 1 nm around the system. Using the gmx genion tool, ions were added to achieve electrostatic neutrality of the system. Long-range electrostatic interactions were treated using the Particle Mesh Ewald (PME) method, with a cutoff distance of 1 nm. Constraints on all bonds were handled using the SHAKE algorithm, and the Verlet leap-frog algorithm was employed with an integration time step of 1 fs.

Prior to the MD simulation, energy minimization was carried out. This involved 3000 steps of steepest descent minimization followed by 2000 steps of conjugate gradient minimization. The energy minimization protocol included the following steps: first, constraining the solute while minimizing the energy of the water molecules; next, constraining the counterions and performing energy minimization; finally, performing unconstrained energy minimization on the entire system.

The MD simulations were conducted under NPT ensemble conditions at a temperature of 310 K and constant pressure, with a total simulation time of 50 ns. During the simulations, the tools g-rmsd, g-rmsf, g-hbond, g-Rg, and g-sasa were used to calculate the root mean square deviation (RMSD), root mean square fluctuation (RMSF), hydrogen bonds (HBonds), radius of gyration (Rg), and solvent-accessible surface area (SASA), respectively.

### Statistical analysis

All data were processed using R version 4.2.1. Pearson’s correlation coefficient was applied to normally distributed data, while Spearman rank correlation was used for non - normally distributed data. Comparisons between two variables were evaluated using the Wilcoxon signed - rank test and the Wilcoxon rank - sum test, respectively. The Kruskal - Wallis test was used to analyze variations among multiple variables. The diagnostic capability of UBD was evaluated using ROC analysis with the ‘pROC’ R package (24). Statistical significance was defined as a p - value less than 0.05, with high significance indicated by a p - value less than 0.0001 (denoted as *p < 0.05, **p < 0.01, ***p < 0.001, and ****p < 0.0001).

## Results

### Expression of UBD in pan-cancer

**Figure 1:**
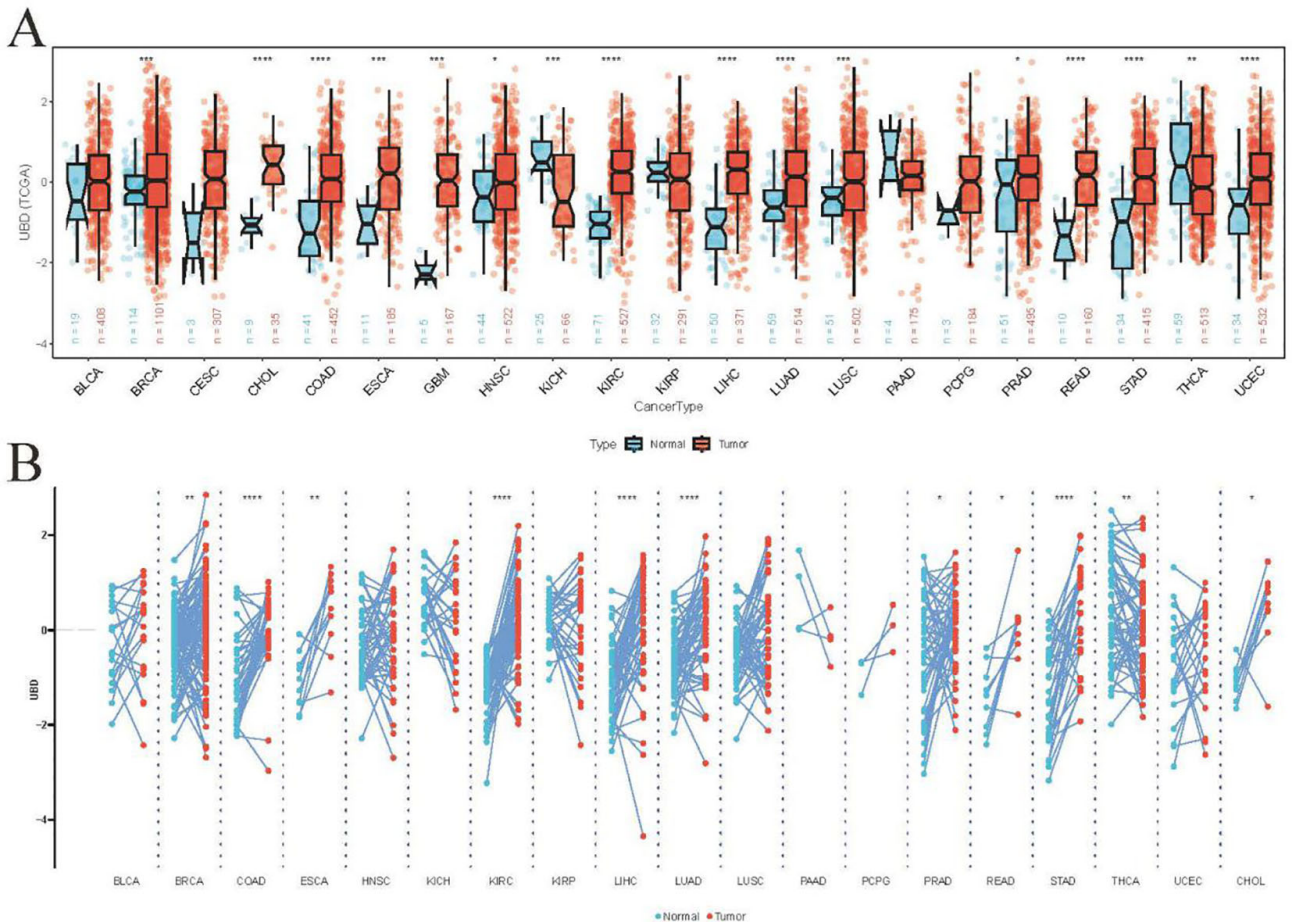
Differential UBD expression across human cancers. **(A)** Comparative analysis of UBD expression levels between tumor tissues and matched adjacent normal tissues across various cancer types in the TCGA cohort. **(B)** Integrated expression profile of UBD demonstrating differential expression between malignant tumors (TCGA dataset) and normal tissues (combined GTEx and TCGA normal samples) across multiple cancer types. * p < 0.05, ** p < 0.01, *** p < 0.001, **** p < 0.0001.

We utilized TCGA cohorts containing both normal and tumor tissue samples to assess the differential expression of UBD across various cancer types (**Figure 1A**). Our analysis revealed that UBD was significantly upregulated in 14 cancer types and downregulated in two malignancies (KICH: Kidney Chromophobe; THCA: Thyroid Carcinoma). In the TCGA cohort, analysis of paired tumor and adjacent normal tissues revealed that UBD was significantly overexpressed in 10 tumor types, while it remained significantly downregulated in THCA (**Figure 1B**).

**Figure 2:**
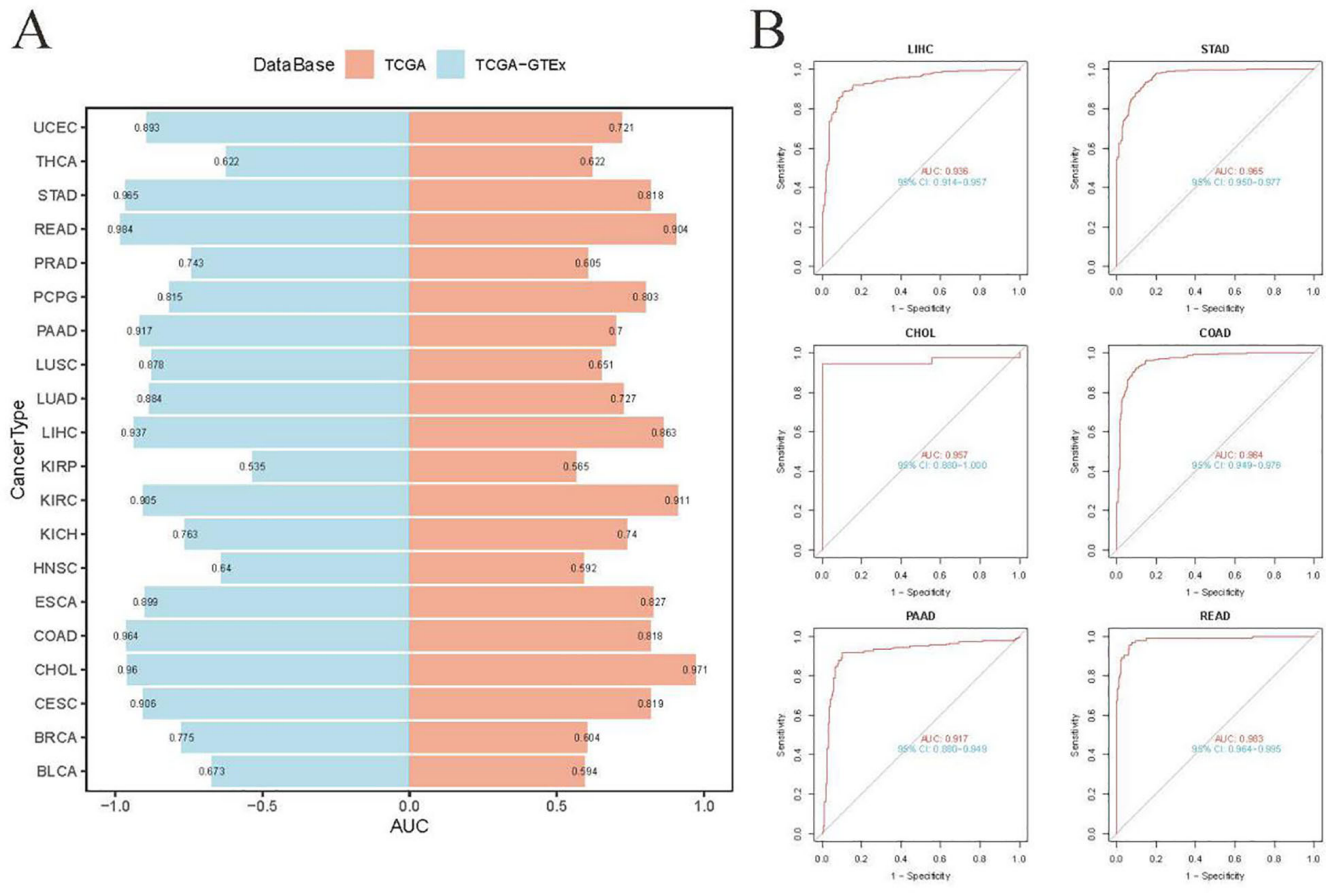
Diagnostic accuracy of UBD across human cancers using ROC curve analysis. **(A)** ROC curve analysis of UBD for distinguishing malignant tumors from normal tissues across multiple cancer types, based on integrated GTEx and TCGA cohorts. **(B)** Diagnostic performance of UBD in gastrointestinal tumors (TCGA dataset), comparing tumor tissues versus combined normal tissues from GTEx and TCGA.

ROC analyses were performed to explore the diagnostic potential of UBD in various types of tumors. The results indicated that the gene expression levels of UBD exhibited strong diagnostic efficacy for gastrointestinal tumors, whether assessed solely in the TCGA cohort or when combined with normal tissue samples from the GTEx database (**Figures 2A, B**).

### UBD in different immune and molecular tumor subtypes

**Figure 3:**
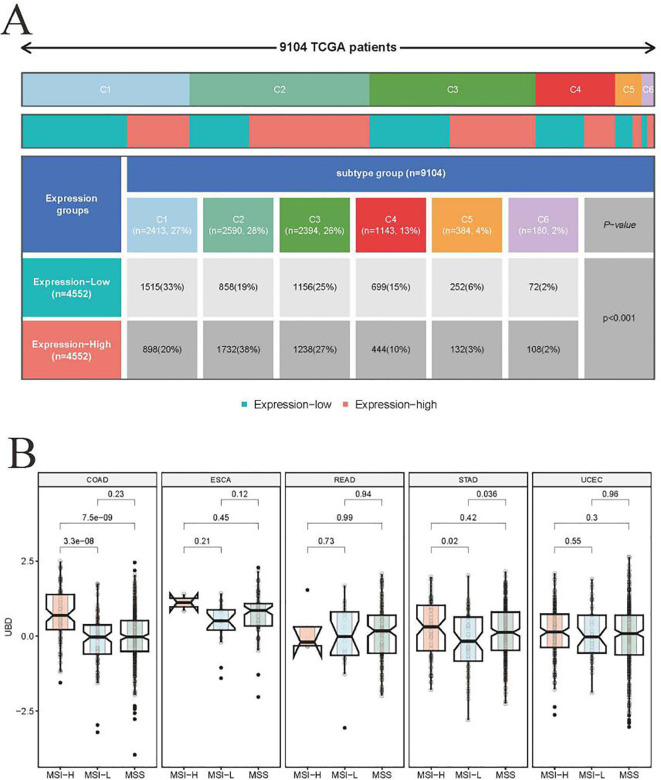
Association of UBD expression with pan-cancer molecular subtypes. **(A)** Stratification of immune subtypes (C1-C6) between high/low UBD expression groups (median mRNA expression cutoff) in the TCGA pan-cancer cohort. **(B)** Comparative analysis of UBD expression profiles across tumors with distinct microsatellite status in cancers (TCGA dataset).

Tumor samples from the TCGA cohort (n = 9,104) were stratified into high and low UBD expression groups based on the median expression value of UBD. These two groups exhibited significantly different immune subtypes (**Figure 3A**). Notably, the proportion of patients with tumors of the C2 (IFN-γ Dominant) subtype was twice as high in the high UBD expression group compared to the low UBD expression group (38% vs. 19%). Additionally, the proportion of patients with tumors of the C1 (Wound Healing) subtype was significantly lower in the high UBD expression group compared to the low UBD expression group (20% vs. 33%). After evaluating the expression levels of UBD in tumors with varying microsatellite instability (MSI) statuses, it was found that UBD expression was significantly associated only with the MSI status in colorectal adenocarcinoma (COAD). In COAD samples, UBD expression levels were significantly higher in those with high microsatellite instability (MSI-H) compared to those with low microsatellite instability (MSI-L) or microsatellite stability (MSS, **Figure 3B**).

Further investigation into the relationship between UBD expression and immune subtypes in COAD revealed a strong association between high UBD expression and the C2 (IFN-γ Dominant) subtype. Specifically, the proportion of the C2 (IFN-γ Dominant) subtype was significantly higher in COAD tumors with high UBD expression compared to those with low UBD expression (32% vs. 7%, **Supplementary Figure 1**).

### Investigating the possible biological roles of UBD in pan-cancer

**Figure 4:**
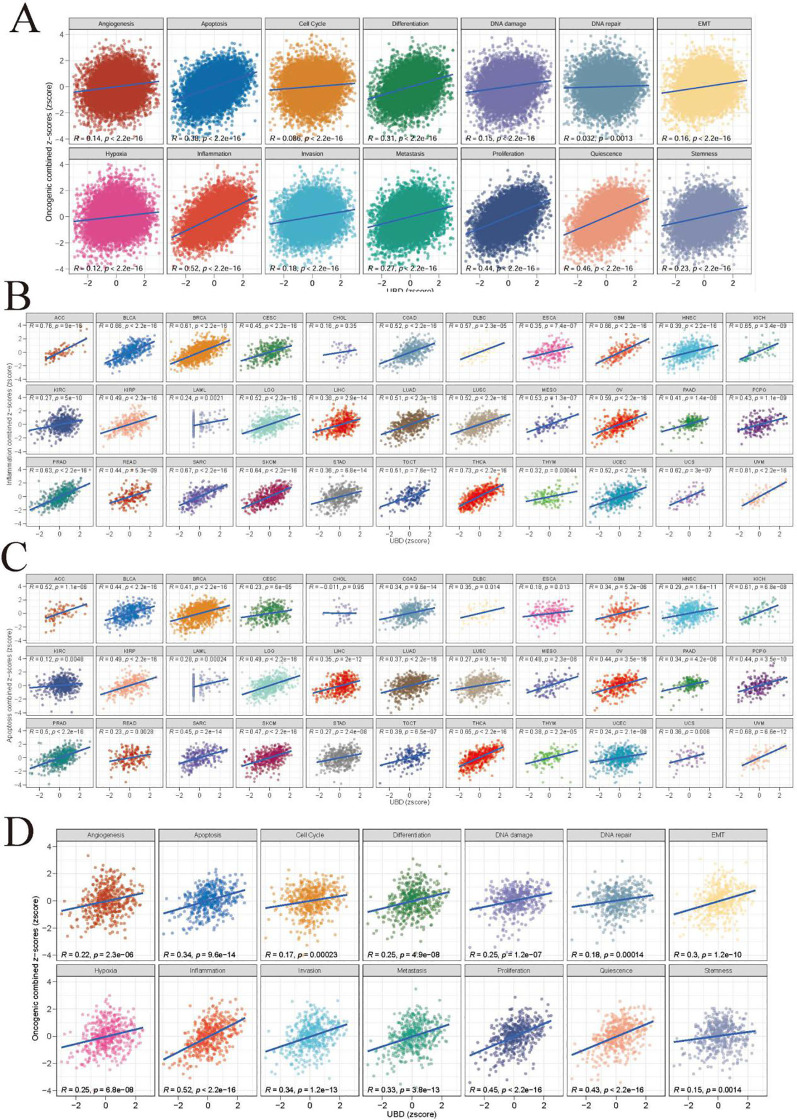
Functional landscape of UBD-associated cancer pathways **(A)** Pan-cancer enrichment analysis of 14 oncogenic pathways correlated with UBD expression (TCGA pan-cancer cohort). Spearman correlation coefficients between UBD expression and inflammation **(B)** and apoptosis **(C)** across 33 cancer types (TCGA dataset). **(D)** In TCGA-COAD cohort, the correlation between UBD expression and 14 oncogenic pathways.

The z-score algorithm proposed by Lee et al. was employed to explore the relationships between UBD and various tumor-related pathways across different cancer types. Previous studies have indicated that the Inflammation and Apoptosis pathways are closely related to the C2 (IFN-γ Dominant) subtype. In this study, the expression levels of UBD exhibited the highest positive correlation with the Inflammation pathway (R = 0.52, **Figure 4A**). Additionally, UBD expression levels also showed a significant positive correlation with the Apoptosis pathway (R = 0.38). We further evaluated the correlations between UBD expression and the Inflammation and Apoptosis pathways across different tumor types (**Figures 4B, C**). In more than two-thirds of the tumor types, UBD expression was significantly positively correlated with the Inflammation pathway, with correlation coefficients exceeding 0.5. Among these, the highest positive correlation was observed in UVM, with a correlation coefficient of R = 0.81. In KICH, THCA, and UVM, the Apoptosis pathway was significantly positively correlated with UBD expression, with correlation coefficients exceeding 0.6.

Given the close association between the C2 (IFN-γ Dominant) subtype and UBD expression in COAD, we further investigated the relationships between UBD and various pathways in COAD (**Figure 4D**). As expected, UBD exhibited the highest positive correlation with the Inflammation pathway (R = 0.52). Additionally, UBD showed a significant positive correlation with the Apoptosis pathway (R = 0.34).

Consistent with the characteristics of the C2 (IFN-γ Dominant) subtype, several inflammation-related Hallmark pathways, including IL2-STAT5 Signaling, IL6-JAK-STAT3 Signaling, Inflammatory Response, Interferon Alpha Response, and Interferon Gamma Response, exhibited significant positive correlations with UBD expression levels (**Supplementary Figure 2**).

### Relationship of UBD with functional proteins in different cancers

**Figure 5:**
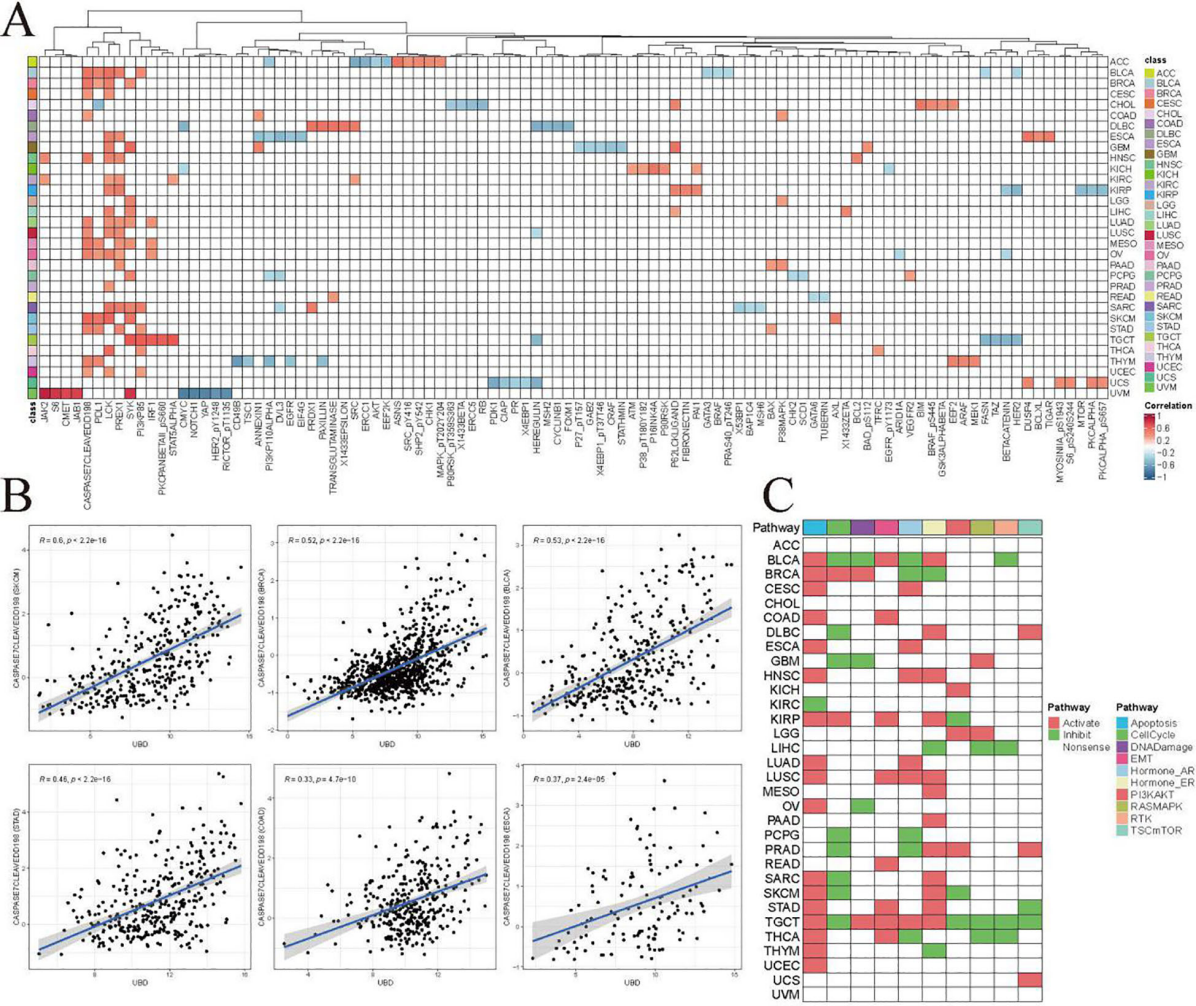
Co-expression patterns of UBD and pathway functional proteins in the TCPA database. **(A)** Additionally, a rank-based association analysis was employed to evaluate the relationship between UBD mRNA levels and the expression of functional proteins measured by Reverse Phase Protein Array (RPPA) in the TCPA database. **(B)** Correlation analysis between Caspase-7 (cleaved at D198) and UBD. **(C)** Proteomic enrichment analysis from the TCPA database presents a heatmap of activated or suppressed pathways in patients with high/low UBD expression.

Additionally, the relationship between UBD mRNA levels and functional proteins expression measured by RPPA in the TCPA database was evaluated using Rank - based association analysis. In UVM, UBD exhibited strong positive correlations with JAK2, S6, CMET, and JAB1. Conversely, UBD showed significant negative correlations with c-MYC, NOTCH1, YAP, HER2 (phosphorylated at Y1248), and RICTOR (phosphorylated at T1135; **Figure 5A**). In testicular germ cell tumors (TGCT), UBD expression was significantly positively correlated with SYK, PI3K p85, IRF1, PKC-panbeta II (phosphorylated at S660), and STAT5α.

Additionally, we identified an intriguing protein cluster composed of CASPASE 7 (cleaved at D198), PDL1, LCK, PREX1, SYK, and PI3K p85. These proteins exhibited significant positive correlations with UBD mRNA expression across more than five cancer types. These proteins may be closely related to the biological functions of UBD.

Caspase-7 (cleaved at D198) is a key effector caspase in the apoptosis cascade, responsible for cleaving and inactivating cellular substrates to execute apoptosis. Caspase-7 (cleaved at D198) exhibited a significant positive correlation with UBD, further suggesting a potential close association between UBD and apoptosis (**Figure 5B**). Proteomic enrichment analysis from the TCPA database indicated that the Apoptosis pathway was in an “activated” state among patients with high UBD expression across 17 cancer types (**Figure 5C**).

### The correlation of UBD expression with tumor immune cell infiltration

**Figure 6:**
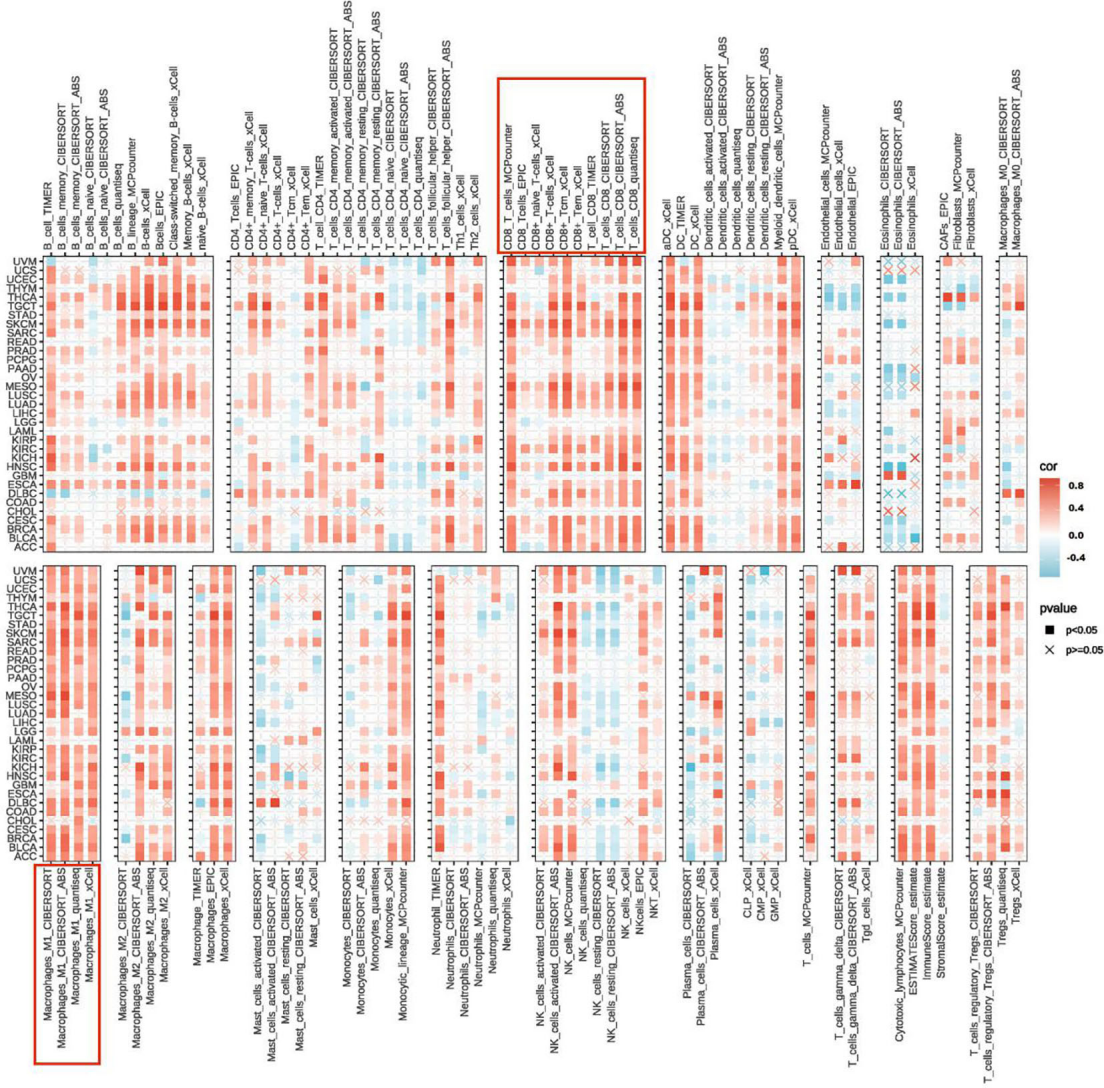
Heatmap illustrating the correlation between UBD expression levels and the infiltration levels of various immune cells.

By integrating various algorithms for calculating immune infiltration scores, we found that the infiltration levels of CD8+ T cells and M1 macrophages were consistently and significantly positively correlated with UBD gene expression across nearly all cancer types (**Figure 6**). The C2 (IFN-γ Dominant) subtype is characterized by a robust immune response with significant infiltration of CD8+ T cells and M1 macrophages. These findings further substantiate the strong association between UBD and the C2 immune subtype of tumors.

### Prognostic value of UBD in pan-cancer

**Figure 7:**
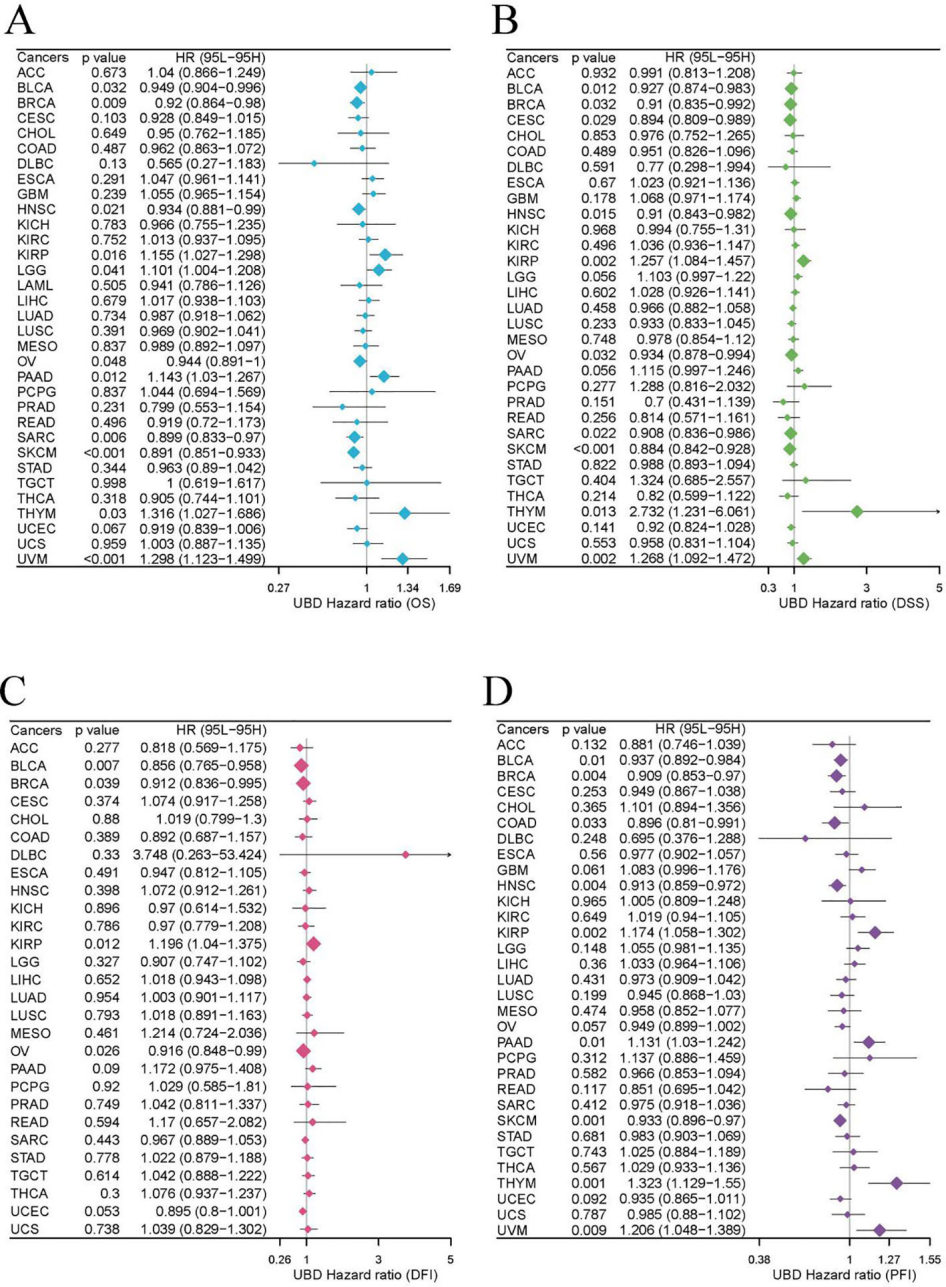
The prognostic value of UBD expression for OS **(A)**, DFI **(B)**, DSS **(C)**, and PFI **(D)**.

Our study evaluated the prognostic value of UBD expression for OS, DFI, DSS, and PFI across various cancers using a univariate Cox regression model (**Figures 7A–D**). As shown in **Figure 7A**, elevated UBD expression was significantly associated with improved OS in breast cancer (BRCA; p=0.009, HR = 0.949) and exerted a protective effect in melanoma (SKCM; p<0.001, HR = 0.891) and sarcoma (SARC; p=0.006, HR = 0.899). Elevated UBD levels are associated with poorer OS in UVM (p<0.001, HR = 1.298), KIRP (p=0.016, HR = 1.155), and PAAD (p=0.012, HR = 1.143).

For DSS, elevated UBD levels were significantly associated with shorter DSS in KIRP (p=0.002, HR = 1.257), thymoma (THYM; p=0.013, HR = 2.732), and UVM (p=0.002, HR = 1.268). Conversely, UBD expression exerted a protective effect in bladder cancer (BLCA; p=0.012, HR = 0.927) and SKCM (p<0.001, HR = 0.884; **Figure 7B**).

Analysis of DFI revealed that high UBD expression was associated with a higher DFI rate in BLCA (p=0.007, HR = 0.856; **Figure 7C**). Additionally, elevated UBD expression correlated with improved PFI in SKCM (p=0.001, HR = 1.323), but with worse PFI in THYM (p=0.001, HR = 1.323) and UVM (p=0.009, HR = 1.206; **Figure 7D**).

To further validate the prognostic utility of UBD, we examined additional datasets containing prognostic information and found consistent results (**Supplementary Figure 3**).

**Figure 8:**
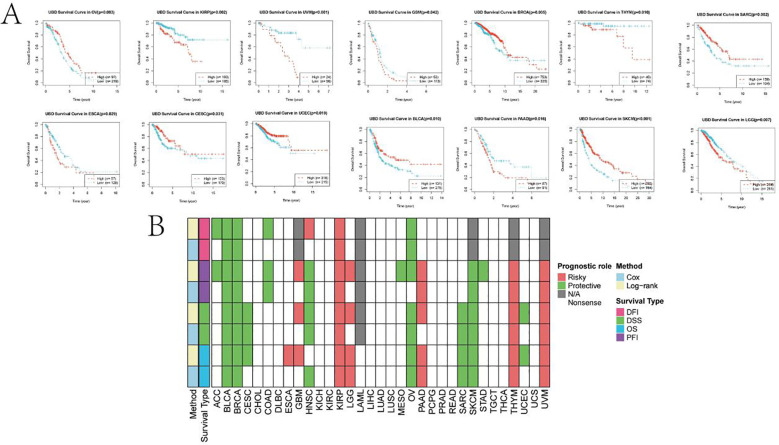
KM analysis of UBD. **(A)** KM survival curves demonstrating UBD prognostic utility in overall survival (OS) across: Protective subgroups: KIRP, UVM, ESCA, THYM, PAAD, and LGG; High-risk subgroups: OV, CESC, UCEC, BRCA, BLCA, SKCM, and SARC. **(B)** Heatmap depicting the pan-cancer prognostic landscape of UBD expression.

Using the R package maxstat to determine the optimal cutoff values for UBD, KM analysis revealed that UBD is a prognostic factor for OS in KIRP, UVM, ESCA, THYM, PAAD, and LGG, acting as a protective factor. In contrast, UBD is associated with poorer prognosis in OV, CESC, UCEC, BRCA, BLCA, SKCM, and SARC (**Figure 8A**). The prognostic value of UBD across pan-cancer was visualized using a heatmap (**Figure 8B**).

### Epigenetic modification of UBD

**Figure 9:**
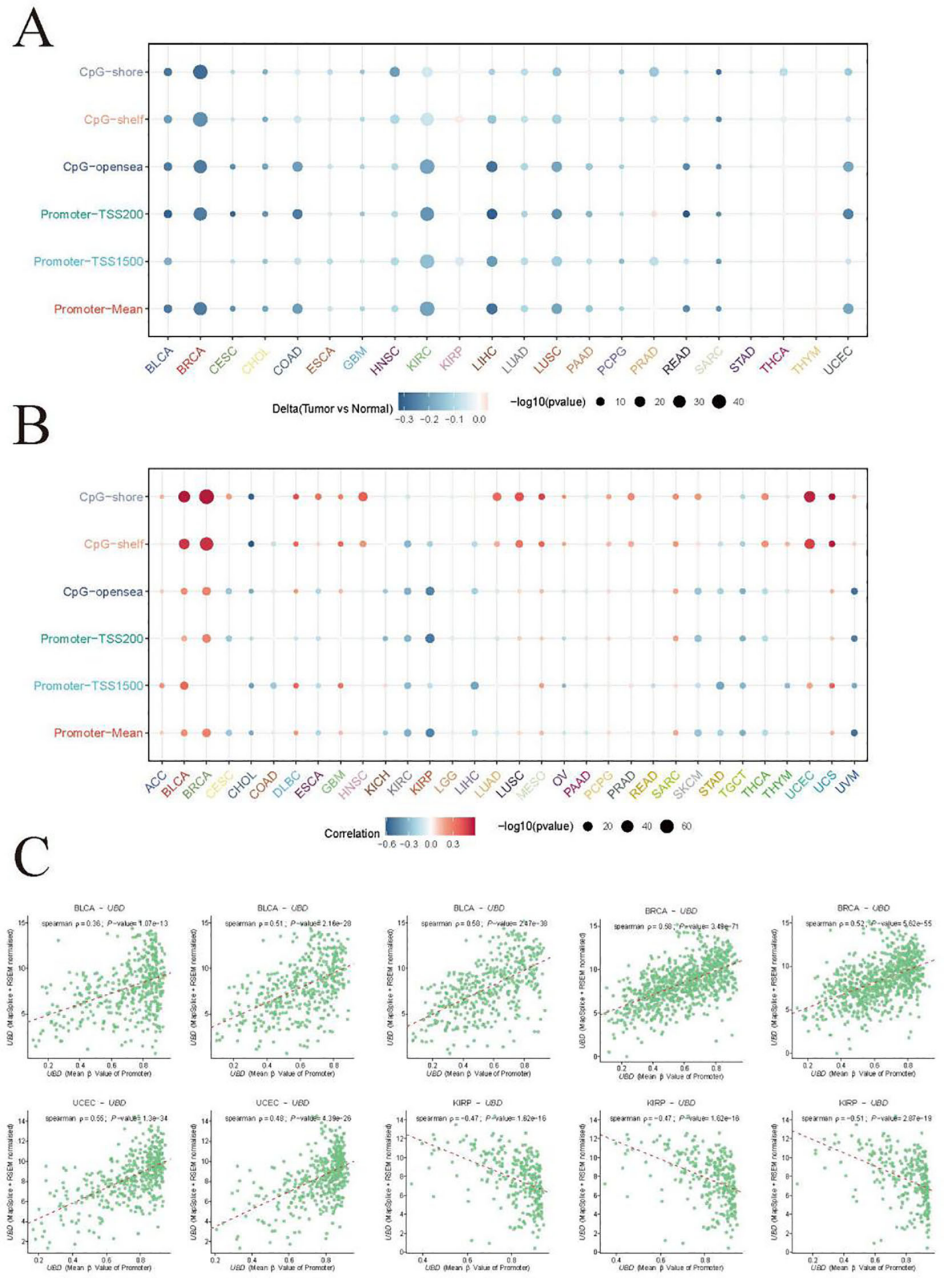
Integrated analysis of UBD gene expression and methylation profiles. **(A)** Regional methylation landscape of UBD CpG sites across differentially methylated regions (DMRs). **(B)** Pan-cancer analysis of associations between UBD promoter methylation and transcriptional activity. **(C)** Correlation analysis of mean β-values in specific DMRs with UBD expression levels in the TCGA-BLCA cohort.

In most cancer types, the methylation levels of UBD across different DNA methylation regions are downregulated in tumors (**Figure 9A**). We visualized the correlation between methylation levels in different regions and UBD gene expression levels across various cancer types using a bubble chart (**Figure 9B**). In BLCA, the mean β values of the TSS1500 (R = 0.36), shelf (R = 0.51), and shore (R = 0.58) methylation regions were significantly positively correlated with UBD gene expression levels (**Figure 9C**). In BRCA, the mean β values of the shelf (R = 0.52) and shore (R = 0.58) methylation regions also exhibited significant positive correlations with UBD gene expression levels. In UCEC, the mean β values of the shelf (R = 0.48), and shore (R = 0.55) methylation regions were significantly positively correlated with UBD gene expression levels. In KIRP, the mean β values of the opensea (R = -0.47), Promoter (R = -0.47), and TSS200 (R = -0.51) methylation regions were significantly positively correlated with UBD gene expression levels.

### Potential roles of UBD in cancer treatment

**Figure 10:**
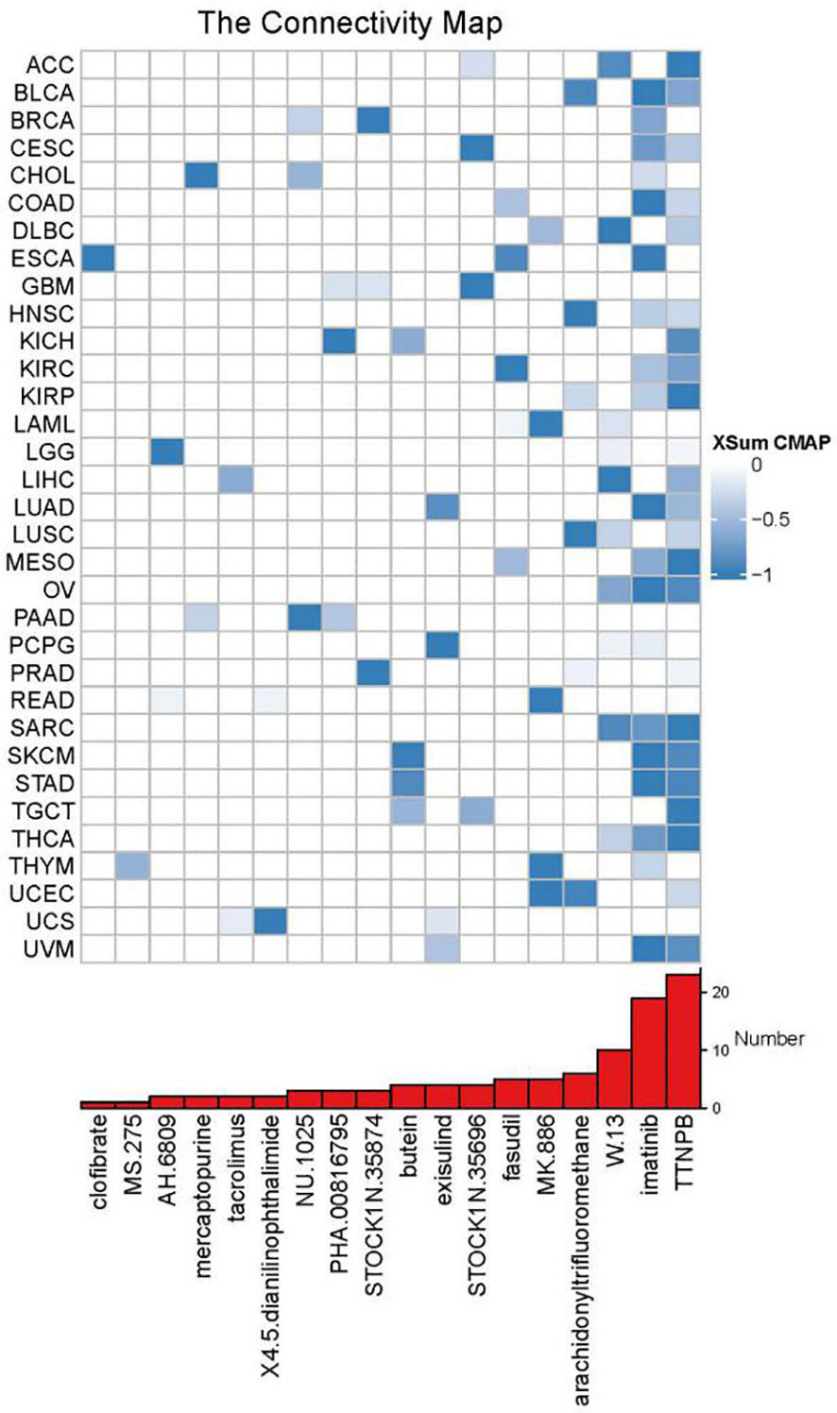
XSum algorithm-based computational screening prioritized chemotherapeutic agents with divergent sensitivity between UBD high- and low-expression subgroups.

Using XSum, CMap can significantly enrich true positive drug-indication pairs through a novel matching algorithm. The lower the XSum relative score, the more likely the drug is to exert anti-tumor effects. When samples were divided into high and low UBD expression groups based on the median value, the results showed that two drugs (imatinib and TTNPB) had therapeutic potential in the high UBD expression group across more than 20 cancer types (**Figure 10**). Imatinib, a tyrosine kinase inhibitor, is commonly used for the treatment of chronic myeloid leukemia (CML) and gastrointestinal stromal tumors (GIST). TTNPB, a retinoic acid receptor (RAR) agonist, has the ability to bind to nuclear RARs with high affinity and can induce G1 cell cycle arrest.

### Molecular docking and molecular dynamics simulations

**Figure 11:**
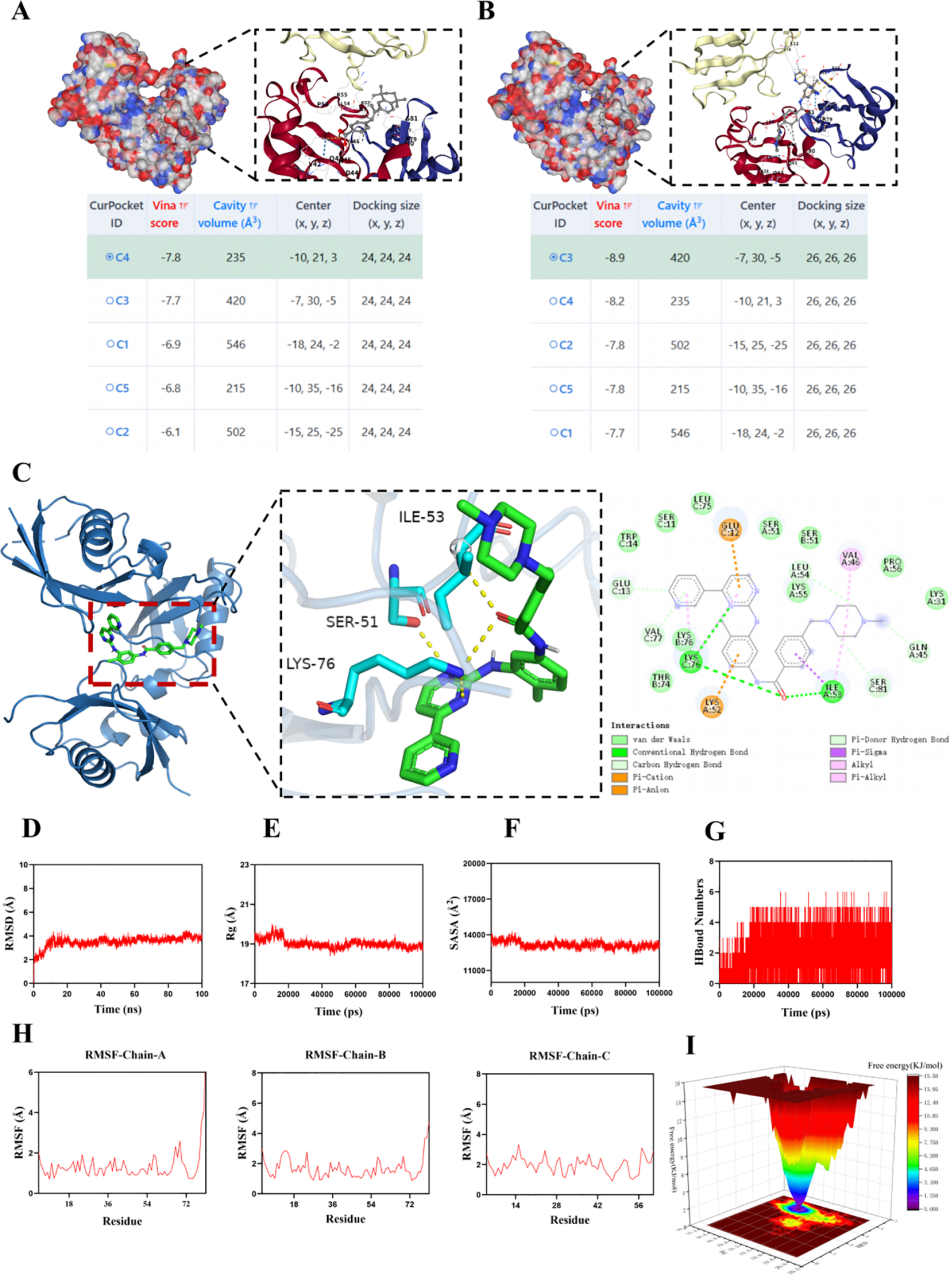
MD simulation of the protein-ligand complex. Information on the five lowest Vina score docking poses for the UBD protein with TTNPB **(A)** and Imatinib **(B)**, along with the docking pose of the lowest Vina score, are provided. **(C)** The detailed docking interaction between UBD protein and Imatinib in conformation C3. **(D)** RMSD values of the protein-ligand complex over time; **(E)** Radius of gyration (Rg) values of the protein-ligand complex over time; **(F)** SASA values of the protein-ligand complex over time; **(G)** HBonds values of the protein-ligand complex over time; **(H)** RMSF values of the amino acid backbone atoms in the protein-ligand complex. **(I)** FEL illustrates the variation trends of free energy, where red regions correspond to high-energy states and blue regions represent low-energy states.

We performed molecular docking of the UBD protein with Imatinib (**Figure 11A**) and TTNPB (**Figure 11B**). We present the five lowest-energy docking poses for each complex, as determined by Vina scoring. The Vina scores for all five docking poses between the UBD protein and Imatinib were lower than –7 kcal/mol, with the C3 pose having the lowest score of –8.9 kcal/mol (**Figure 11C**). Therefore, compared to TTNPB, Imatinib shows better docking affinity with UBD. Accordingly, we conducted MD simulations on the molecular docking complex of Imatinib and UBD.

The root mean square deviation (RMSD) is a good indicator of the conformational stability of proteins and ligands, as well as a measure of the deviation of atomic positions from their initial positions. A smaller deviation indicates greater conformational stability. Therefore, RMSD was used to evaluate the equilibration of the simulation system. As shown in **Figure 11D**, the complex system reached equilibrium after 10 ns and eventually fluctuated around 3.7 Å. Thus, Imatinib exhibits high binding stability with UBD. The radius of gyration (Rg) is a measure that describes overall structural changes and can be used to characterize the compactness of a protein structure. A greater variation in Rg indicates a more expanded system. Further analysis revealed that the complex system exhibited slight fluctuations during the simulation and gradually stabilized. This suggests that conformational changes occurred in the Imatinib–UBD complex during the simulation (**Figure 11E**). The solvent-accessible surface area (SASA) is a metric used to evaluate the surface area of a protein. In this simulation, the SASA between Imatinib and UBD was calculated (**Figure 11F**). The results show that the complex system exhibits slight fluctuations and gradually stabilizes over time. This demonstrates that the binding of the small molecule affects the local microenvironment and leads to a certain degree of change in SASA. Hydrogen bonds play an important role in the binding of ligands to proteins. The number of hydrogen bonds between Imatinib and UBD during the molecular dynamics simulation is shown in **Figure 11G**. The number of hydrogen bonds ranged from 0 to 6, and in most cases, the complex formed approximately 5 hydrogen bonds, indicating strong and favorable hydrogen bonding interactions between the ligand and the target protein. The root mean square fluctuation (RMSF) indicates the flexibility of amino acid residues within a protein. As shown in **Figure 11H**, the RMSF values for this complex are relatively low (mostly below 0.9-2.7 Å), indicating that the residues have lower flexibility and higher stability. The free energy landscape (FEL) illustrates the free energy distribution calculated based on RMSD and RG during molecular dynamics simulations of protein-ligand interactions. Color gradients are employed to represent free energy levels, transitioning from red (high energy) to blue (low energy). The dynamic simulation process is depicted in **Figure 11I**.

In summary, the complex exhibits stable binding and has favorable hydrogen bonding interactions. Therefore, the binding interaction between Imatinib and UBD is strong and effective.

### UBD expression in ESCA: experimental validation and clinical prognosis

RT-qPCR analysis revealed a significant upregulation of UBD gene expression in ESCA tumor tissues, with expression levels positively correlating with tumor staging (**Supplementary Figure 4A**). The highest UBD expression was observed in stage IV ESCA. WB analysis demonstrated upregulated UBD protein expression levels in ESCA tumor samples, with expression levels progressively increasing in correlation with advancing tumor stages (**Supplementary Figures 4B, C**). IF assays further confirmed elevated UBD expression in ESCA tumor tissues (**Supplementary Figure 4D**).

A total of 30 paraffin-embedded tissue blocks from ESCA patients were included in this study. The clinical characteristics of the patients are summarized in **Supplementary Table 1**. Based on the IHC-based AOD values of UBD, the 30 enrolled ESCA patients were stratified into high, moderate, and low UBD groups using tertiles (**Supplementary Figure 4E**). The low UBD group exhibited the highest proportion of stage I (40%) and stage II ESCA (50%) cases and the lowest proportion of stage III cases (10%), while all stage IV patients were exclusively categorized into the high UBD group. OS was defined as the interval from the date of initial diagnosis to the occurrence of all-cause mortality or the last follow-up contact. Survival data were primarily obtained through the hospital’s electronic health records system. In instances where death documentation was unavailable, survival status verification was performed via structured telephone interviews. Kaplan-Meier curve analysis demonstrated that patients in the high UBD group exhibited significantly poorer OS outcomes (**Supplementary Figure 4F**).

### UBD promotes the malignant phenotypes of proliferation and migration in esophageal cancer via the TP53 signaling pathway

Previous results indicated that esophageal cancer patients with high UBD expression had poorer prognoses, prompting us to further explore the biological functions of UBD in esophageal cancer. First, we established a stable TE-11 cell line overexpressing UBD. Both UBD mRNA and protein levels were upregulated by more than 5-fold (**Supplementary Figures 5A, B**). Plate colony formation assay (**Supplementary Figure 5C**) and CCK8 cell proliferation curves (**Supplementary Figure 5D**) both indicated that the proliferative capacity of TE-11 cells overexpressing UBD was significantly enhanced. Wound healing assay (**Supplementary Figure 5E**) and Transwell migration assay (**Supplementary Figure 5F**) both demonstrated that the migratory ability of TE-11 cells overexpressing UBD was significantly increased.

To investigate the underlying mechanisms responsible for these observed differences in proliferation and migration, we performed transcriptome sequencing on TE-11 cells transduced with empty vector control lentivirus (n=3) and TE-11 cells overexpressing UBD (n=3).

Using thresholds of absolute fold change >2 and false discovery rate (FDR) <0.05, volcano plots showed that 221 genes were upregulated and 328 genes were downregulated in UBD-overexpressing TE-11 cells (**Supplementary Figures 5G, H**). The heatmap displays the top 20 upregulated and downregulated genes (**Supplementary Figure 5I**). Pathway enrichment analysis using all differentially expressed genes was performed with Metascape, and the results indicated that the TP53 signaling pathway and TP53-related pathways (such as cell cycle regulation and cellular senescence) were significantly enriched in TE-11 cells overexpressing UBD (**Supplementary Figure 5J**). To further validate our findings, we used Western blotting to examine key proteins related to TP53 and the cell cycle, including c-myc, cyclin B1, CDK1, CDK4, p53, and p21. The expression levels of c-myc, cyclin B1, CDK1, and CDK4 were upregulated in TE-11 cells overexpressing UBD, while the expression levels of p53 and p21 were significantly downregulated (**Supplementary Figure 5K**). These results are consistent with our enrichment analysis. Therefore, the promotion of esophageal cancer proliferation and migration by UBD may be mediated through the TP53 signaling pathway.

## Discussion

This study represents the first comprehensive pan-cancer analysis of UBD, elucidating its multifaceted roles across malignancies. Our findings demonstrate that UBD is aberrantly expressed in a cancer-specific manner, with significant upregulation in gastrointestinal and hepatic cancers, contrasting with downregulation in THCA and KICH. Such tissue-specific dysregulation aligns with UBD’s induction by pro-inflammatory cytokines like IFN-γ and TNF-α, suggesting its role as a molecular nexus between chronic inflammation and tumor progression. The observed dual prognostic impact of UBD—protective in SKCM and SARC yet detrimental in UVM and PAAD—highlights context-dependent functionalities, potentially governed by TME dynamics. For instance, UBD’s pro-apoptotic effects in SKCM may counteract tumor growth, whereas its genomic destabilizing properties in UVM could exacerbate malignancy. Previous studies have demonstrated that UBD directly interacts with IRE1α, thereby modulating the activation of its downstream JNK signaling pathway and regulating cytokine-induced apoptosis (25).

A pivotal discovery is UBD’s strong association with the C2 (IFN-γ-dominant) immune subtype, characterized by robust CD8+ T cell and M1 macrophage infiltration (26–28). This aligns with prior reports implicating UBD in MHC-I antigen presentation and immune evasion (6). Mechanistically, UBD’s correlation with inflammation-related pathways (e.g., IL6-JAK-STAT3, interferon response) and apoptosis effectors like Caspase-7 underscores its dual role in modulating immune surveillance and cell death (29–32). While previous studies have provided some supportive evidence, it should be emphasized that our findings only demonstrate a strong association between UBD and the C2 (IFN-γ-dominant) immune subtype across pan-cancer analyses—a causal relationship has not been established. Further validation through *in vitro* and *in vivo* studies is warranted in future research. Furthermore, our proteomic profiling revealed a co-expression pattern among UBD, Caspase-7, PD-L1, and JAK2, which may plausibly be linked to UBD’s strong association with the C2 (IFN-γ-dominant) immune subtype. However, it should be noted that this study did not provide definitive mechanistic validation of these interactions through direct assays such as co-immunoprecipitation or knockdown-rescue experiments. However, accumulating evidence from previous studies has suggested potential functional links between UBD and Caspase-7, PD-L1, and JAK2. For example, Previous studies have revealed that UBD upregulates PD-L1 expression in tumors through activation of the PI3K/AKT/mTOR signaling pathway, independent of its ubiquitin-like modification function (33). This finding demonstrates UBD’s potential to promote tumor immune evasion by elevating PD-L1 levels, highlighting its promise as a novel therapeutic target to enhance the efficacy of cancer immunotherapy. Nava Reznik et al. showed that JAK2 serves as a key upstream regulator of UBD expression. Studies have demonstrated that the JAK2 inhibitor AZ960 significantly downregulates UBD expression induced by pro-inflammatory cytokines such as IFNγ, TNFα, and IL-6. JAK2 promotes the phosphorylation of STAT1/3/5 proteins, facilitating their nuclear translocation where they function as transcription factors to directly or indirectly enhance UBD transcription (31). Thus, inhibition of JAK2 effectively reduces UBD expression, indicating that the JAK-STAT signaling pathway plays a central role in the regulation of UBD.

Functional enrichment and proteomic analyses revealed UBD’s interplay with oncogenic pathways. The z-score algorithm highlighted UBD’s strong correlation with inflammation and apoptosis, while RPPA data implicated JAK2 and S6 kinase as downstream effectors. Nava et al. elucidated the signaling pathways governing UBD expression under pro-inflammatory conditions that typify TMEs (31). Employing a high-throughput phenotypic transcriptional reporter screen with a mechanistically annotated compound library, the investigators identified AZ960 - a selective JAK2 kinase inhibitor - as a potent suppressor of cytokine-induced UBD expression. Notably, this downregulation occurred independently of canonical NFκB signaling. Through systematic genetic knockdown validation, JAK2 was established as a primary transcriptional regulator of UBD, with subsequent mechanistic studies implicating STAT1/3/5 phosphorylation cascades in mediating this regulatory axis. This work not only delineates the JAK-STAT-UBD signaling module in inflammation-driven malignancies but also provides AZ960 as a pharmacological probe for dissecting UBD’s pathophysiological roles through targeted expression modulation. These findings resonate with UBD’s reported role in destabilizing tumor suppressors like p53, suggesting a broader regulatory network influencing proliferation and survival (7). Intriguingly, UBD-high tumors exhibited sensitivity to imatinib, a tyrosine kinase inhibitor, and TTNPB, a retinoid agonist, hinting at therapeutic vulnerabilities exploitable in combinatorial regimens (7).

The inconsistent prognostic role of UBD—protective in cancers like SKCM and SARC but risky in UVM and PAAD—likely arises from its context-dependent functions within distinct TMEs. In immunogenic tumors (e.g., SKCM), UBD upregulation is linked to the C2 (IFN-γ-dominant) immune subtype, characterized by CD8+ T cell infiltration and pro-apoptotic activity, promoting anti-tumor responses. Conversely, in immunosuppressive TMEs (e.g., UVM, PAAD), UBD may facilitate immune evasion via PD-L1 upregulation and p53 degradation, driving progression. Thus, UBD’s dual impact reflects a balance between its pro-apoptotic versus oncogenic degradation roles, dictated by the immune and molecular context of each cancer.

Additionally, we partially revealed the relationship between UBD and the malignant phenotype of esophageal cancer for the first time using overexpressed esophageal cancer cell lines and transcriptomic sequencing. UBD enhances the proliferation and migration of esophageal cancer cells through the TP53 signaling pathway. These results are consistent with the prognosis information we collected on esophageal cancer: patients with high UBD expression have poorer prognoses. In a study by Hongbin Su and colleagues, UBD significantly enhanced the proliferative capacity of colorectal cancer (CRC) cells by promoting p53 degradation (7). Mechanistically, UBD directly binds to p53 and regulates its ubiquitin-proteasome-dependent degradation, markedly shortening the half-life of the p53 protein, thereby downregulating p21 expression and upregulating cell cycle regulators such as Cyclin D1, Cyclin E, and CDK2/4/6, thus driving the cell cycle progression. This finding aligns with the classical mechanism where ubiquitin-like proteins dynamically regulate target protein stability through an E1-E2-E3 enzyme cascade (7). Notably, in the study by Hongbin Su et al., UBD-induced tumor growth in nude mice was dependent on the downregulation of p53 expression, indicating that its oncogenic effects are closely related to the inactivation of the p53 signaling pathway (7).

Despite these advances, limitations warrant consideration. First, The utilization of GTEx normal tissues as a reference for differential expression analysis may introduce confounding variability due to discrepancies in donor characteristics, preservation methods, and collection protocols. These factors could potentially skew tumor-normal comparisons, especially in cancer types where matched normal samples are scarce within TCGA. Future studies with larger cohorts of meticulously matched normal tissues are warranted to refine these observations. Then, while bulk RNA sequencing provide robust transcriptional insights, spatial resolution of UBD’s expression within tumor niches remains unexplored. Then, the findings in this study were experimentally validated in esophageal carcinoma; however, their generalizability to a pan-cancer context remains limited.

Future research should prioritize elucidating UBD’s post-translational modifications and interactome to identify novel binding partners. Additionally, exploring UBD’s synergy with immune checkpoint inhibitors in C2-subtype cancers could unveil strategies to enhance immunotherapy responsiveness. Longitudinal studies tracking UBD expression during treatment may further refine its utility as a dynamic biomarker.

## Conclusions

In summary, this study reveals that UBD is aberrantly expressed across multiple cancer types and may serve as a potential prognostic biomarker. Molecular docking results suggest that imatinib is a promising therapeutic compound targeting UBD. In esophageal cancer, UBD overexpression promotes cell proliferation and migration by modulating the TP53 signaling pathway. These findings highlight UBD as a promising oncogenic biomarker and therapeutic target, particularly in the context of immunotherapy and precision medicine.

## Data Availability

The original contributions presented in the study are included in the article/**Supplementary Material**. Further inquiries can be directed to the corresponding author.
