# Supplementary material for: Pan-cancer landscape of UBD/FAT10 and experimental validation in esophageal carcinoma
Source: Front Oncol. 2025 Nov 19;15:1615898. doi: 10.3389/fonc.2025.1615898 (PMC12672329; doi:10.3389/fonc.2025.1615898)
Supplement: Supplementary file 1 [file DataSheet1.docx]

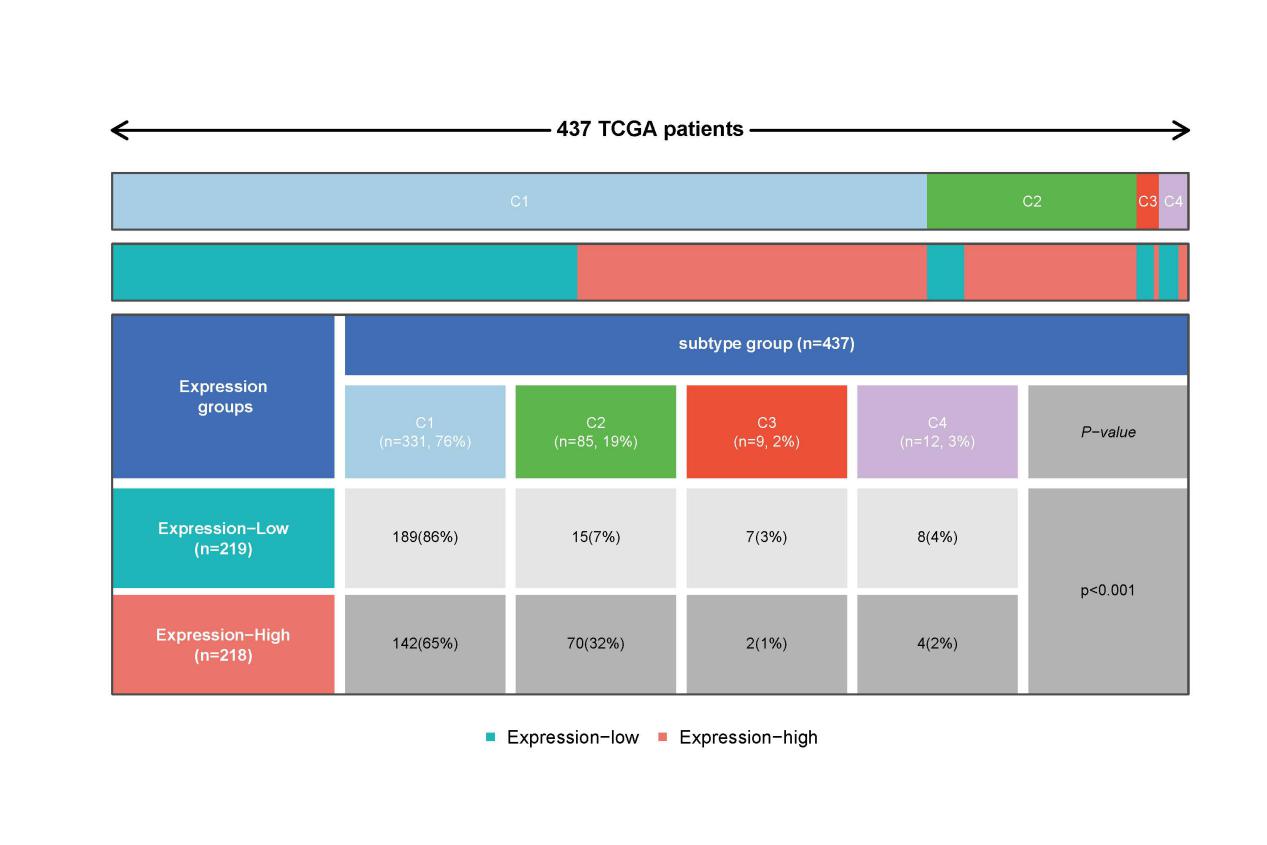


**Supplementary Figure 1. Stratification of immune subtypes (C1-C6) between high/low UBD expression groups (median mRNA expression cutoff) in the TCGA-COAD cohort.**


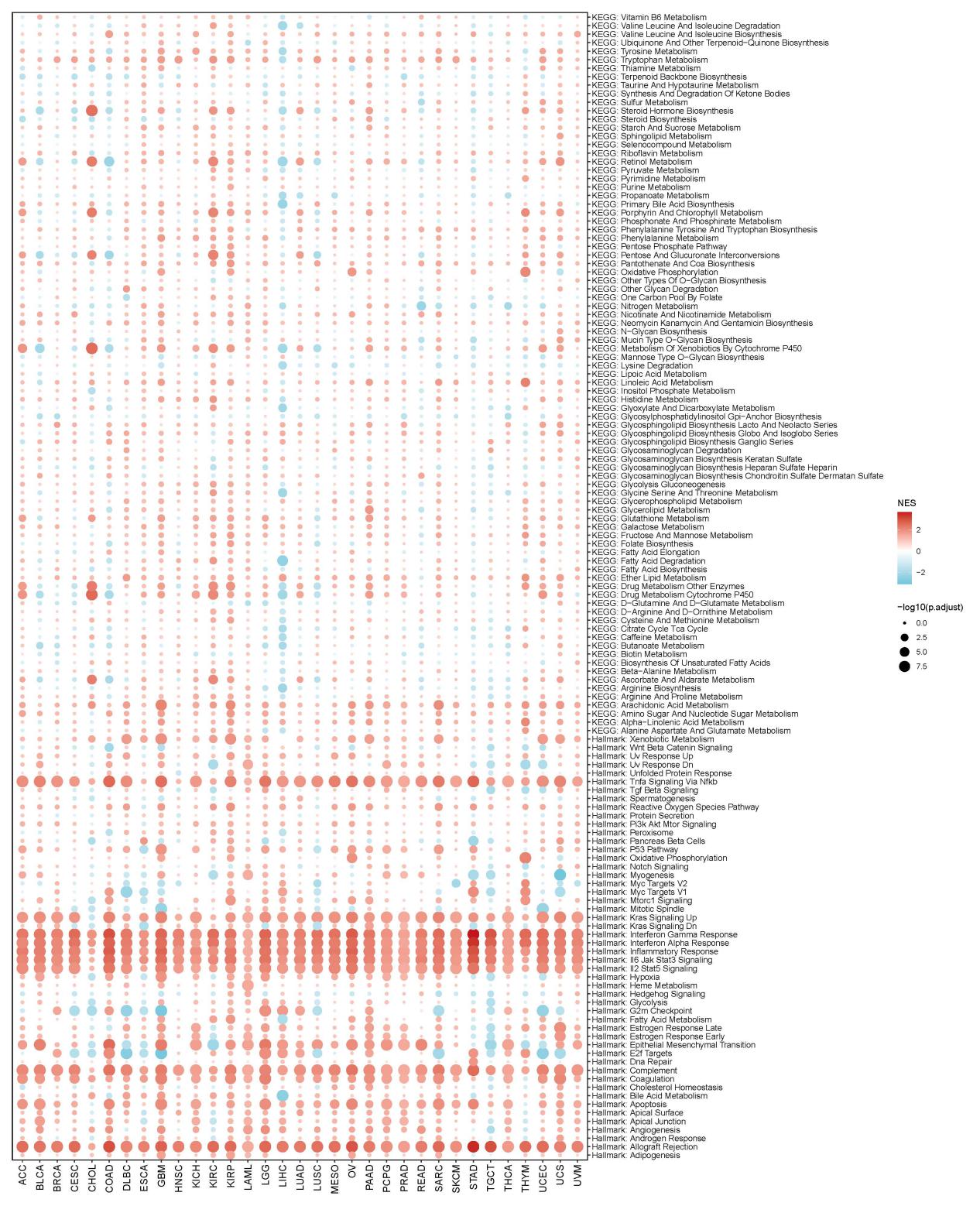


**Supplementary Figure 2: Dot plot illustrating the correlation analysis between UBD expression levels and enrichment scores of different KEGG and Hallmark pathways across 33 types of cancer.**


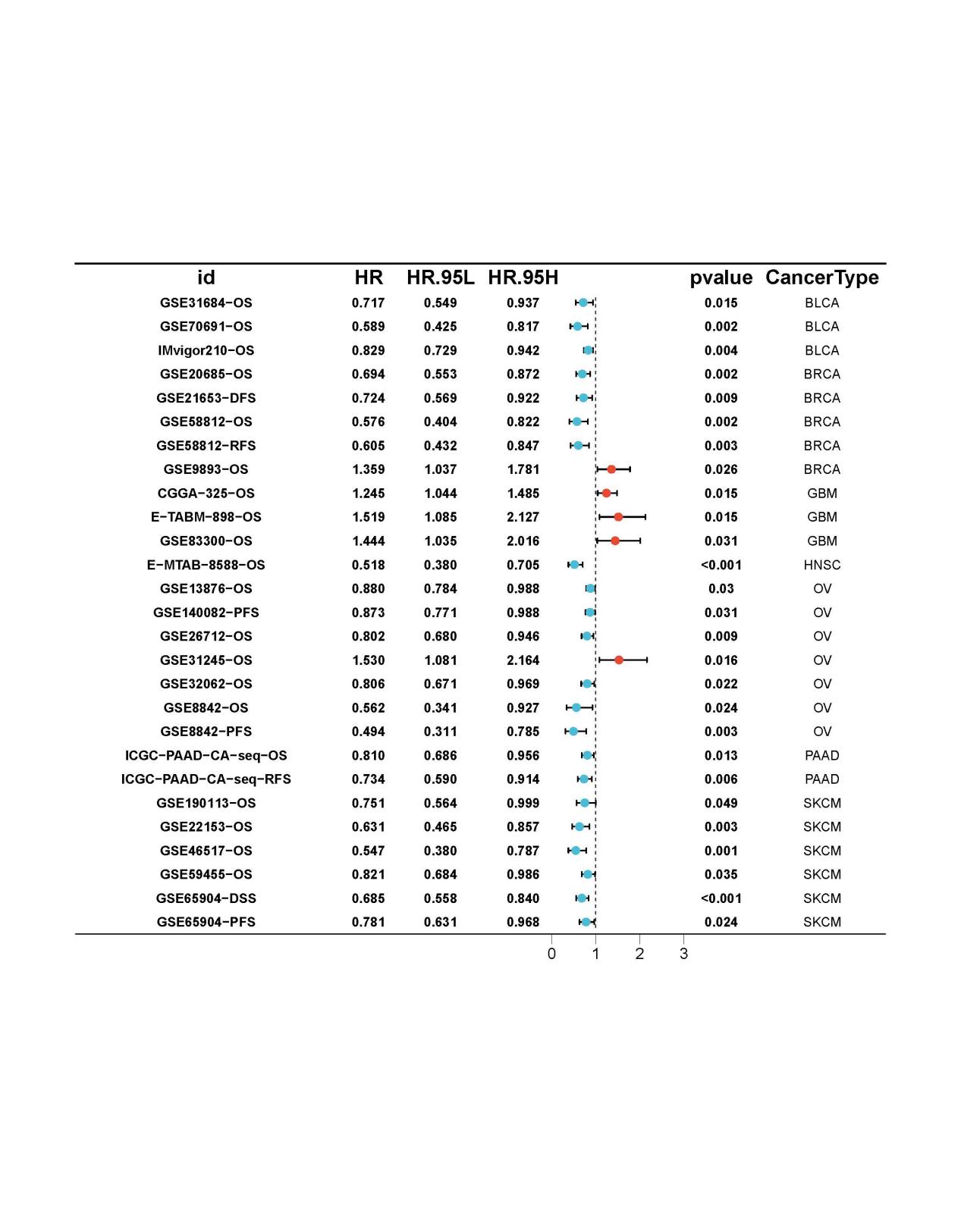


**Supplementary Figure 3: Independent validation of the prognostic utility of UBD.**

**
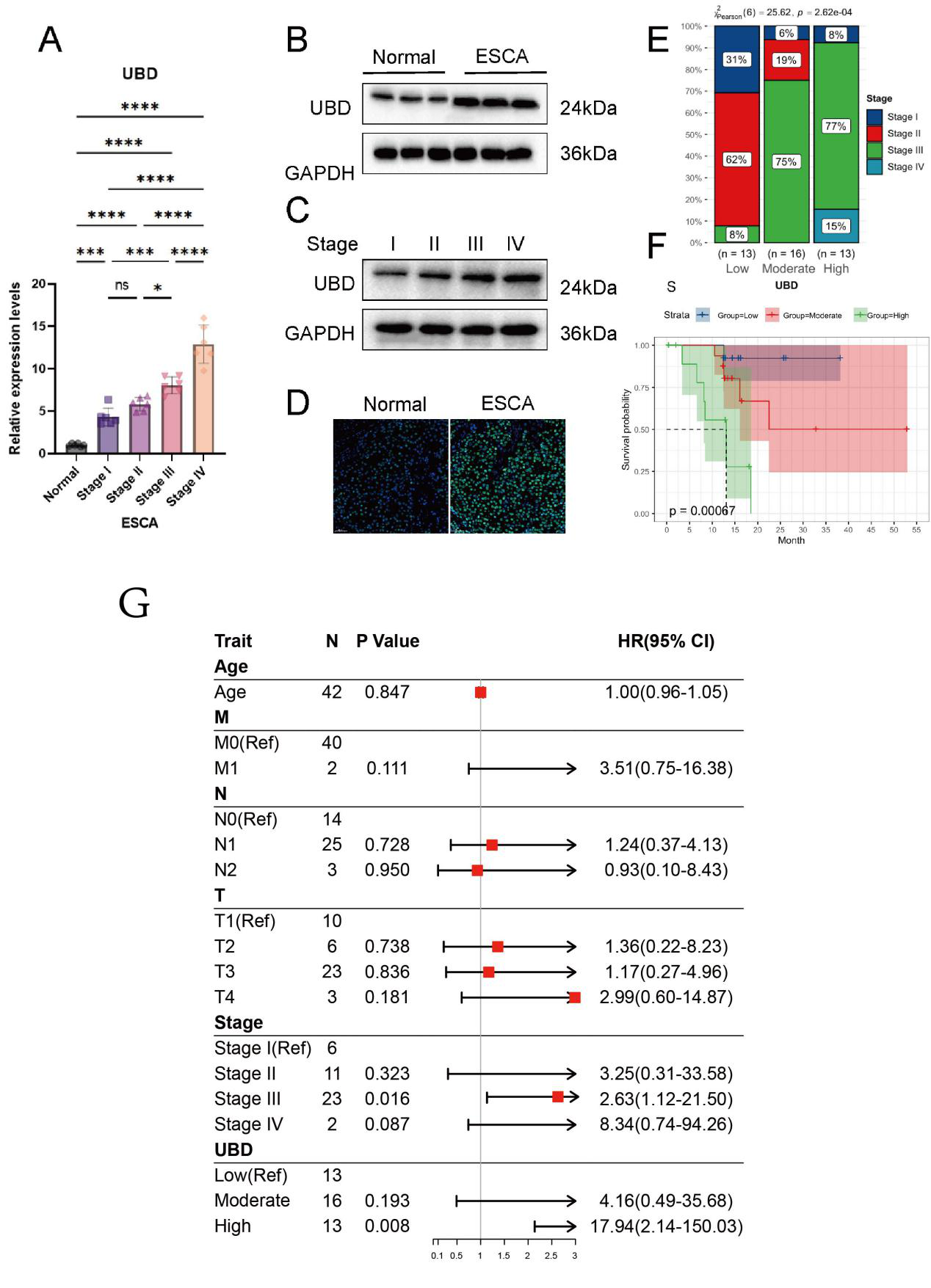
**

**Supplementary Figure 4.‌ Experimental Validation and Clinical Prognosis of UBD in ESCA. ‌(A)‌ RT-qPCR analysis of UBD gene expression levels between adjacent non-tumor tissues and ESCA specimens across different TNM stages.**

**‌(B)‌ WB analysis of UBD protein expression levels in adjacent non-tumor tissues versus ESCA tissues.‌(C)‌ WB analysis of UBD protein expression across ESCA specimens stratified by TNM stage.‌(D)‌ IF analysis of UBD in adjacent non-tumor and ESCA tissues. Nuclei were counterstained with DAPI (blue); UBD signal is shown in green.‌(E)‌ Proportion of ESCA patients at distinct TNM stages within high, moderate, and low UBD expression groups.‌(F)‌ KM survival curves comparing OS among ESCA patients stratified into high, moderate, and low UBD expression groups. ‌(G)‌ Forest Plot of Multivariable Cox Regression.**


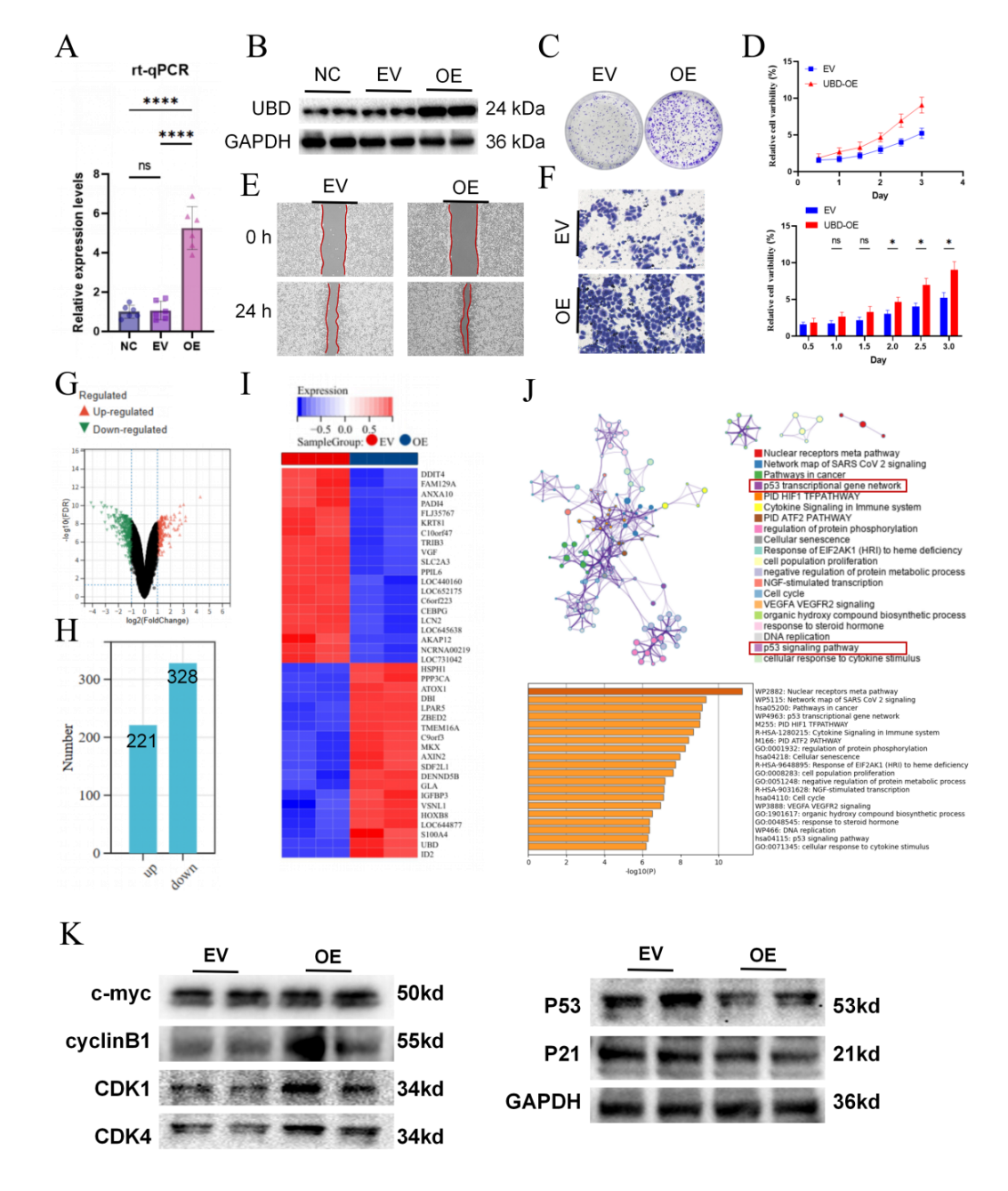


**Supplementary Figure 5. Overexpression of UBD upregulates the proliferation and migration phenotypes in esophageal cancer via the TP53 signaling pathway.Differences in UBD gene expression levels (A) and protein expression levels (B) between TE-11 cells transduced with control lentivirus and those overexpressing UBD. Plate colony formation assays (C) and CCK8 cell proliferation curves (D) comparing control and UBD-overexpressing TE-11 cells. Wound healing assays (E) and Transwell migration assays (F) comparing control and UBD-overexpressing TE-11 cells. Volcano plot showing differential analysis results (G). The number of upregulated and downregulated differentially expressed genes in UBD-overexpressing TE-11 cells (H). Heatmap displaying the top 20 upregulated and downregulated differentially expressed genes (I). Enrichment analysis of differentially expressed genes (J). Western blot analysis of TP53 and cell cycle-related protein expression levels in control and UBD-overexpressing TE-11 cells (K).EV: Empty Vector Control; OE: Overexpression Group.**

**Supplementary Table 1: The clinical characteristics of the patients**

| Variable | Overall, N = 42^1^ | | Low, N = 13^1^ | Moderate, N = 16^1^ | High, N = 13^1^ | p-value^2^ |
| --- | --- | --- | --- | --- | --- | --- |
| **Gender** |  | |  |  |  | 0.3 |
| female | 6 (14%) | | 3 (23%) | 3 (19%) | 0 (0%) |  |
| male | 36 (86%) | | 10 (77%) | 13 (81%) | 13 (100%) |  |
| **Age** | 55 (48, 66) | | 55 (48, 67) | 52 (44, 59) | 56 (55, 61) | 0.2 |
| **M** |  | |  |  |  | 0.2 |
| M0 | 40 (95%) | | 13 (100%) | 16 (100%) | 11 (85%) |  |
| M1 | 2 (4.8%) | | 0 (0%) | 0 (0%) | 2 (15%) |  |
| **N** |  | |  |  |  | 0.014 |
| N0 | 14 (33%) | | 8 (62%) | 5 (31%) | 1 (7.7%) |  |
| N1 | 25 (60%) | | 4 (31%) | 11 (69%) | 10 (77%) |  |
| N2 | 3 (7.1%) | | 1 (7.7%) | 0 (0%) | 2 (15%) |  |
| **T** |  | |  |  |  | 0.008 |
| T1 | 10 (24%) | | 6 (46%) | 1 (6.2%) | 3 (23%) |  |
| T2 | 6 (14%) | | 4 (31%) | 1 (6.2%) | 1 (7.7%) |  |
| T3 | 23 (55%) | | 3 (23%) | 11 (69%) | 9 (69%) |  |
| T4 | 3 (7.1%) | | 0 (0%) | 3 (19%) | 0 (0%) |  |
| **Stage** |  | |  |  |  | <0.001 |
| Stage I | 6 (14%) | | 4 (31%) | 1 (6.2%) | 1 (7.7%) |  |
| Stage II | 11 (26%) | | 8 (62%) | 3 (19%) | 0 (0%) |  |
| Stage III | 23 (55%) | | 1 (7.7%) | 12 (75%) | 10 (77%) |  |
| Stage IV | 2 (4.8%) | | 0 (0%) | 0 (0%) | 2 (15%) |  |
| **Site** |  | |  |  |  | 0.002 |
| Lower | 17 (40%) | | 1 (7.7%) | 11 (69%) | 5 (38%) |  |
| Middle | 23 (55%) | | 12 (92%) | 5 (31%) | 6 (46%) |  |
| Upper | 2 (4.8%) | | 0 (0%) | 0 (0%) | 2 (15%) |  |
| ^1^n (%); Median (IQR) | |  |  |  |  |  |
| ^2^Fisher's exact test; Kruskal-Wallis rank sum test | |  |  |  |  |  |
